# Global Vertical Distribution of Water Vapor on Mars: Results From 3.5 Years of ExoMars‐TGO/NOMAD Science Operations

**DOI:** 10.1029/2022JE007231

**Published:** 2022-09-26

**Authors:** S. Aoki, A. C. Vandaele, F. Daerden, G. L. Villanueva, G. Liuzzi, R. T. Clancy, M. A. Lopez‐Valverde, A. Brines, I. R. Thomas, L. Trompet, J. T. Erwin, L. Neary, S. Robert, A. Piccialli, J. A. Holmes, M. R. Patel, N. Yoshida, J. Whiteway, M. D. Smith, B. Ristic, G. Bellucci, J. J. Lopez‐Moreno, A. A. Fedorova

**Affiliations:** ^1^ Department of Complexity Science and Engineering Graduate School of Frontier Sciences The University of Tokyo Kashiwa Japan; ^2^ Royal Belgian Institute for Space Aeronomy Brussels Belgium; ^3^ NASA Goddard Space Flight Center Greenbelt MD USA; ^4^ Department of Physics American University Washington DC USA; ^5^ Space Science Institute Boulder CO USA; ^6^ Instituto de Astrofísica de Andalucía Glorieta de la Astronomia Granada Spain; ^7^ Institute of Condensed Matter and Nanosciences Université catholique de Louvain Louvain‐la‐Neuve Belgium; ^8^ School of Physical Sciences The Open University Milton Keynes UK; ^9^ Tohoku University Sendai Japan; ^10^ Centre for Research in Earth and Space Science York University Toronto ON Canada; ^11^ Istituto Nazionale di Astrofisica Rome Italy; ^12^ Space Research Institute (IKI) Moscow Russia

## Abstract

We present water vapor vertical distributions on Mars retrieved from 3.5 years of solar occultation measurements by Nadir and Occultation for Mars Discovery onboard the ExoMars Trace Gas Orbiter, which reveal a strong contrast between aphelion and perihelion water climates. In equinox periods, most of water vapor is confined into the low‐middle latitudes. In aphelion periods, water vapor sublimated from the northern polar cap is confined into very low altitudes—water vapor mixing ratios observed at the 0–5 km lower boundary of measurement decrease by an order of magnitude at the approximate altitudes of 15 and 30 km for the latitudes higher than 50°N and 30–50°N, respectively. The vertical confinement of water vapor at northern middle latitudes around aphelion is more pronounced in the morning terminators than evening, perhaps controlled by the diurnal cycle of cloud formation. Water vapor is also observed over the low latitude regions in the aphelion southern hemisphere (0–30°S) mostly below 10–20 km, which suggests north‐south transport of water still occurs. In perihelion periods, water vapor sublimated from the southern polar cap directly reaches high altitudes (>80 km) over high southern latitudes, suggesting more effective transport by the meridional circulation without condensation. We show that heating during perihelion, sporadic global dust storms, and regional dust storms occurring annually around 330° of solar longitude (*L*_S_) are the main events to supply water vapor to the upper atmosphere above 70 km.

## Key Points

- We present global vertical distributions of water vapor in the Mars atmosphere from the observations collected for 3.5 years
- We confirm a strong contrast between aphelion and perihelion water vapor vertical distributions
- We reveal that water vapor sublimated from the northern polar cap is confined into very low altitudes during the aphelion periods

## 1 Introduction

Water is always present as water vapor in the atmosphere of Mars even though the abundance is relatively small. After the discovery of water vapor in the Mars atmosphere by ground‐based observations (Spinrad et al., 1963), many measurements by telescopes and Mars orbiters have been performed to investigate the water cycle on Mars (e.g., Clancy et al., 1996, 2017; Farmer et al., 1977; Fedorova et al., 2006; Fouchet et al., 2007; Maltagliati et al., 2008; Smith, 2002, 2004; Smith et al., 2009). They revealed the global picture of the seasonal water cycle: water vapor is sublimated from the polar caps and transported into middle‐low latitudes in the local spring‐summer season, and water vapor is condensed back onto the polar caps in the local autumn‐winter seasons. They also revealed that water ice clouds at low‐latitudes in aphelion periods and at middle‐high latitudes in local autumn/winter, play an important role in the atmospheric water cycle on Mars (e.g., Clancy et al., 1996; Smith, 2004). However, these views were obtained based mostly on the measurements of water vapor abundances integrated over the total column of the atmosphere, and the information of vertical distribution were limited.

The first insight into the vertical profiles of water vapor was provided by a series of microwave ground‐based observations at Kitt Peak National Observatory and the Very Large Array (VLA) (Clancy et al., 1996), which indicated water vapor vertical distributions have a strong contrast between aphelion and perihelion periods. They argued that water vapor saturation typically occurs at much lower altitudes (below 10 km) during northern spring‐summer, and that the Mars atmosphere exhibits a distinct aphelion climate with a global aphelion cloud belt and ∼20 K lower global atmospheric temperatures relative to perihelion. They also suggested that the north‐south transport of water vapor by the Hadley circulation at aphelion period is blocked by the presence of water ice clouds (so‐called “Clancy effect”), which causes north‐south asymmetries of Mars water vapor and the residual polar ice caps. With a general circulation model (GCM), Montmessin et al. (2004) demonstrated that transport of water vapor from the north to the south at aphelion period is indeed blocked by the formation of the clouds, however, water can still be transported but in the form of ice clouds. More recently, Kahre et al. (2020) and Haberle et al. (2019) argued that modest southward transport of water in this season is primarily associated with eddies in the lower atmosphere, below the aphelion cloud belt.

Spectroscopy for Investigation of Characteristics of the Atmosphere of Mars (SPICAM) onboard Mars Express (MEX) was the first instrument which was able to directly obtain vertical profiles of water vapor from orbit by solar occultation measurements (Maltagliati et al., 2011, 2013; Fedorova et al., 2018, 2021). Maltagliati et al. (2011) found that water vapor in the Mars atmosphere is frequently present in excess of saturation (“super‐saturation”) in the northern spring‐summer season. The occurrence of super‐saturation has been an important argument since saturation conditions control the seasonal and spatial variabilities of Mars atmospheric water (e.g., Clancy et al., 1996; Farmer et al., 1977). Maltagliati et al. (2013) showed that high abundances of water vapor are present in the middle atmosphere in the southern spring season, more than previously considered in GCMs. They also suggested strong interactions between water vapor and aerosols, based on a tentatively defined correlation between water vapor and aerosols profiles that was observed. Fedorova et al. (2018) found that the amount of water vapor in the middle atmosphere in the southern spring‐summer season is even more increased during the global dust storm, based on SPICAM measurement in Mars year (MY) 28. Fedorova et al. (2021) confirmed the different behaviors of the water vapor in the middle atmosphere during aphelion and perihelion periods (“quiet” in the former, and “dramatic” in the latter such as short‐term variations, detached layers, etc.) from the SPICAM measurements for 8 Mys (MY 27–34).

Two new spectrometers onboard ExoMars Trace Gas Orbiter (TGO)—Nadir and Occultation for Mars Discovery (NOMAD; Vandaele et al., 2018) and Atmospheric Chemistry Suite (ACS; Korablev et al., 2018)—are able to monitor the water vapor vertical distribution through a whole MY with much better coverage. Thanks to the TGO orbit optimized for solar occultation measurements, NOMAD and ACS can observe 24 occultations per day at a maximum (average 5–7 observations per day) and obtain a latitudinal map of water vapor from ∼0 to 30 km up to 120 km above the areoid with a sampling of ∼1 km for every ∼20° of *L*_S_. With such an unprecedented vertical sampling, NOMAD and ACS have revealed many new details of the vertical distribution of water vapor (Alday et al., 2021; Aoki et al., 2019; Belyaev et al., 2021; Fedorova et al., 2020; Vandaele et al., 2019; Villanueva et al., 2021). Vandaele et al. (2019) and Aoki et al. (2019) showed a rapid and significant increase of water vapor in the middle atmosphere (40–100 km) during global dust storm and regional dust storm that occurred in MY 34. A theoretical study with GCM simulation explained that atmospheric heating induced by an increase of the dust abundances due to the storm prevents water from condensing as ice clouds and allows water vapor to extend into the middle atmosphere (Neary et al., 2020). Villanueva et al. (2021) and Fedorova et al. (2020) showed that significant amounts of water vapor are also transported into the upper atmosphere during the southern summer season especially at higher latitudes greater than 60°S, which can reach at least around 120 km (Belyaev et al., 2021). This is consistent with predictions by GCMs (e.g., Daerden et al., 2019; Lefèvre et al., 2004; Shaposhnikov et al., 2019), which suggests that meridional circulation in that particular period around perihelion transports water vapor into such high altitudes. Fedorova et al. (2020) showed that supersaturation of water is common at high altitudes during the dust storms and during perihelion season when clouds are present, which implies that more water is able to be sustained at the high altitudes and can escape to space.

To date, the analysis of water vapor vertical distributions by the TGO measurements has been focused on or limited to the perihelion season. This study presents the water vapor vertical profiles retrieved from the 3.5 years measurements of the TGO/NOMAD taken from April 2018 to September 2021, which covers nearly two full Mars years. With this extended data set, we can investigate new details of water vapor vertical distributions during aphelion periods in MY 35 and 36 when key phenomenon to understand water cycle on Mars occur, such as sublimation of water vapor from the northern polar cap and transport of water from the northern to the southern hemisphere. We can also investigate the perihelion periods in a year with global dust storm (MY 34) and non‐global dust storm (MY 35) with this extended data set. The details of the data set and analysis are described in Section 2. General seasonal variations of the retrieved water vapor vertical distributions are presented in Section 3. Seasonal evolutions of the latitudinal variations of the retrieved water vapor vertical distributions are presented in Section 4. In Section 5, comparisons between the retrievals from NOMAD and ACS are discussed. In Section 6, water vapor saturation ratios are discussed with the atmospheric temperatures predicted by Global Environmental Multiscale Mars model (GEM‐Mars, Daerden et al., 2019; Neary et al., 2020).

## 2 Data Set and Analysis

### 2.1 1. Instrument—NOMAD Onboard TGO

NOMAD is a spectrometer onboard ExoMars TGO, operating between 0.2 and 4.3 μm (Vandaele et al., 2018). It has three spectral channels and two out of three are operating in the near infrared ranges. One is dedicated for solar occultation measurements operating at 2.3–4.3 μm (Solar Occultation [SO] channel) and the other one is capable of nadir, solar occultation, and limb measurements at 2.3–3.8 μm (Limb Nadir and solar Occultation (LNO) channel). The third channel covers ultraviolet and visible spectral ranges between 200 and 650 nm (Ultraviolet and Visible Spectrometer [UVIS] channel). The main optics of the infrared channels (the SO and LNO) are an echelle grating and Acousto Optical Tunable Filter (AOTF) (Neefs et al., 2015), which enables us to achieve relatively high spectral resolution (λ/dλ ∼ 17,000 and ∼10,000 for the SO and LNO channels, respectively). Each solar occultation measurement by NOMAD is performed every ∼1 s and a set of spectra is acquired approximately at each 1 km from the surface to 250 km altitude. During each solar occultation measurement for ∼1 s, the AOTF can change the observing diffraction orders instantaneously so that the SO channel can measure five or six different diffraction orders. Nominal operation with NOMAD SO channel returns four spectra for each diffraction order taken simultaneously but corresponding to different pixels on the detector (i.e., scanning at slightly different location in spatial domain; hereafter, we call then as spectra collected at different “bins”). The advantages of the NOMAD SO channel compared to the near‐infrared spectrometers onboard previous missions are its higher spectral resolution and the TGO orbit optimized for solar occultation measurements. These allow us to investigate vertical profiles of main atmospheric constituents such as CO_2_, CO, H_2_O, and HDO (Aoki et al., 2019; Vandaele et al., 2019; Villanueva et al., 2021) together with aerosols (Liuzzi et al., 2020, 2021) and ozone from the UVIS channel (Khayat et al., 2021; Patel et al., 2021) and sensitive searches of trace gases (such as CH_4_, C_2_H_4_, C_2_H_6_, H_2_CO, HCl, HCN, HO_2_, H_2_S, N_2_O, OCS; Aoki et al., 2021; Korablev et al., 2019, 2021; Knutsen et al., 2021; Liuzzi, Villanueva, Trompet, et al., 2021). In this study, we analyze the data taken by the SO channel.

Early works on calibration of the NOMAD SO channel from the first in‐flight data and laboratory measurements are summarized in Liuzzi et al. (2019) and Thomas et al. (2022). Villanueva et al. (2022) presents the up‐to‐date calibration of the NOMAD SO channel, which has been extensively improved from the earlier studies. The major updates presented in Villanueva et al. (2022) are new characterization of (a) Instrumental line shape (ILS), and (b) AOTF transfer function. Villanueva et al. (2022) showed that ILS is not an ideal Gaussian function but can be characterized by a sum of two Gaussian functions, whose parameters for each pixel are determined from the measurements of the Mars atmosphere. They also extensively investigate the AOTF transfer function based on the in‐flight calibration measurements of the Sun that have been acquired for 3 years of the operation at Mars orbit. These calibration updates allow us to improve the quality of the molecular retrievals.

The data reduction of the NOMAD SO spectra is described in detail in Aoki et al. (2019) and Trompet et al. (2022). Transmittances are obtained by dividing the spectra measured through the Mars atmosphere by the reference solar spectrum that is extrapolated from those recorded outside the atmosphere. This division removes systematic instrumental effects. The calculated transmittances are compared with synthetic spectra calculated by a radiative transfer model to perform inversion analysis and retrieve water vapor abundances.

### 2.2 Data Analysis

**Figure 1:**
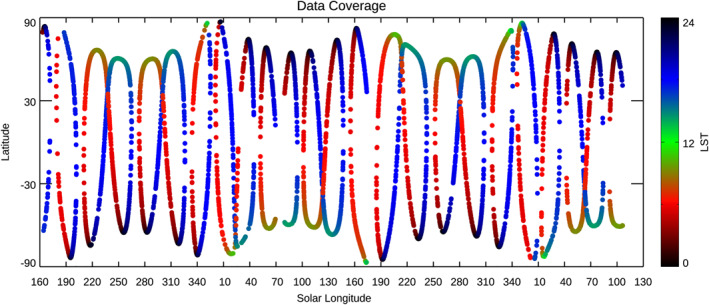
Solar longitude (*x*‐axis) and latitude (*y*‐axis) of the solar occultation measurements taken from 21 April to 30 September 2021 by Trace Gas Orbiter/Nadir and Occultation for Mars Discovery used in this study. The color denotes the local time of the measurements.

We use the measurements taken at diffraction orders 134 (3,011–3,035 cm^−1^) and 136 (3,056–3,080 cm^−1^). It is known that the curve of growth is not linear when the total optical depth along the line of sight exceeds unity (hereafter, “saturated”). Because the intensities of the water lines in these spectral ranges are moderate (S ∼ 10^−21^ cm^−1^/(molecule cm^−2^)), the lines are not heavily saturated even in the near surface spectra so that water vapor vertical profiles can be retrieved from 0 km up to ∼90 km. Stronger water lines are available in order 168 (3,775–3,805 cm^−1^) and order 169 (3,798–3,828 cm^−1^), which can be used to investigate water vapor abundances at higher altitudes up to ∼120 km (e.g., Aoki et al., 2019). In this study, measurements taken from 21 April 2018 to 30 September 2021 have been analyzed, corresponding to *L*_S_ = 162° in Mars Year (MY) 34 and *L*_S_ = 106° in MY 36, which covers ∼2 Mars years. In this period, a total of 5,472 profiles of water vapor were retrieved from the selected diffraction orders. Figure 1 shows the latitudinal and local time (LT) coverages of these occultations. As shown in this figure, the latitude shifts from orbit to orbit, at the same time, the longitudinal coverage is distributed all over the planet. It demonstrates that this data set allows us to obtain a latitudinal map for every ∼20° of *L*_S_. The LT is generally around 6 a.m. or 6 p.m. (±2 hr) except the measurements at the higher latitudes.

**Figure 2:**
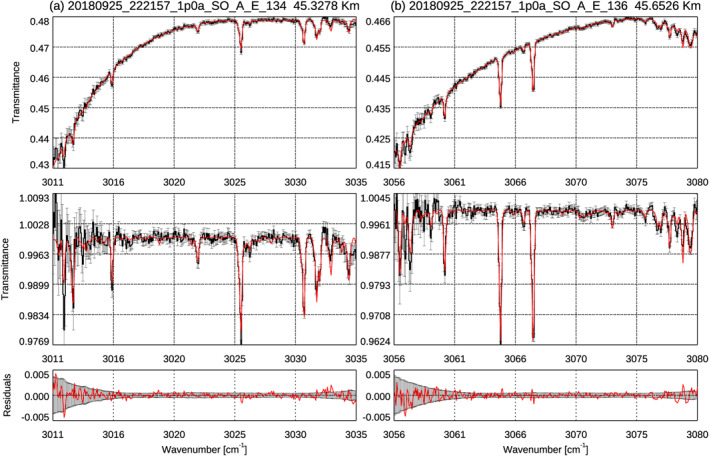
Example of spectra measured by Trace Gas Orbiter/Nadir and Occultation for Mars Discovery (black curves) with orders 134 (a) and 136 (b). These spectra are measured around 45 km above areoid on 25 September 2018 (*L*_S_ = 257° in MY 34) at latitude = 64°S, longitude = 7°W, LT = 23 hr. The red curves show the best‐fit synthetic spectra. The top and middle panels show the original transmittances and those after removal of the continuum, respectively. The bottom panels illustrate the residuals of the top panel in red and the instrumental noise level in black. The unit of the *x*‐axis is wavenumber (cm^−1^). There is a very good agreement between volume mixing ratios retrieved from orders 134 and 136 (87.9 ± 3.3 and 88.0 ± 2.3 ppmv, respectively).

The retrieval is conducted with the same methodology as the previous water vapor and HCl studies (Aoki et al., 2019, 2021; Vandaele et al., 2019). The retrieval is performed by Atmospheric Spectra Inversion Modular Utility Tools (ASIMUT)—Asimut Lidort VLidort (ALVL) radiative transfer and inversion code (Vandaele et al., 2006), which is able to consider spherical geometry for solar occultation measurements. We performed the retrievals until the total optical depth along the line of sight is less than 3.0, that is, the mean transmittance of the spectra is less than 0.05. H_2_O and CO_2_ molecular absorptions are taken into account in the radiative transfer calculation. H_2_O absorption coefficients are calculated by line‐by‐line method with the spectroscopic parameters corrected for CO_2_‐rich atmosphere (Régalia et al., 2019). The line parameters of CO_2_ are based on HITRAN2016 (Gordon et al., 2017) where the air‐broadening coefficients are replaced by the self‐broadening values. For each spectrum, temperature, pressure, and CO_2_ volume mixing ratio of the simulated atmosphere are obtained from the GEM‐Mars (Daerden et al., 2019; Neary & Daerden, 2018) which takes into account the effects of the global dust storm in MY 34 (Neary et al., 2020). The vertical coordinate is defined by height above the MGM1025 areoid (Mars geoid) (Lemoine et al., 2001). The retrievals are performed using the optimal estimation method (Rodgers, 2000) for each spectrum for each tangential altitude and for each bin independently. Spectrum at each altitude is processed as independent measurements, and the H_2_O column density along the line‐of‐sight (LOS) are retrieved by assuming constant volume mixing ratio along the LOS. This method is effective for the NOMAD SO data set because sounding latitudes and longitudes (and thus temperature and pressures) change dramatically during an occultation. In the retrieved vertical profiles of water vapor, that most of the information comes from the sounded tangent altitude (about 70% of the slant number density integrated over the LOS is within 4 km from the tangent height), thus the retrieved local H_2_O abundances at the tangential altitudes of the measurements can be considered as its vertical profiles (Aoki et al., 2019). The retrieved H_2_O abundances in each bin, that is, at tangential height, are finally averaged on a common vertical grid with an interval of 1 km. The averages are weighted by their standard errors (weighted average). The standard deviation of the retrieved H_2_O abundances within 1 km altitudes provides a realistic relative uncertainty of the final H_2_O abundances, which is ∼10% of the retrieved water vapor volume mixing ratio, in average. The error increases the upper/lower boundary of the vertical profiles where the signal‐to‐noise ratio decreases. The detection of water vapor more than 3‐sigma confidence is considered and discussed as valid results in this paper. Figure 2 shows an example of the NOMAD spectra and best‐fit synthetic spectra at diffraction order 134 (Figure 2a) and order 136 (Figure 2b). The agreements between the measured and synthetic spectra are quite good and within the instrumental noise. In the early work in Aoki et al. (2019), we de‐weighted the corresponding spectral ranges if a water line was saturated. This is not necessary for the present study because the water lines in orders 134 and 136 are not heavily saturated.

In addition to the error primary due to the instrumental noise, the following three sources of errors should be taken into account: (a) uncertainty in the atmospheric temperature used in the calculation of radiative transfer (i.e., calculation of absorption coefficients of gases), (b) uncertainty in the atmospheric density to calculate volume mixing ratio, and (c) uncertainty due to the imperfect knowledge of the NOMAD instrumental functions such as the AOTF transfer function. The temperature in the radiative transfer calculation relies on the prediction from GEM‐Mars for each observing geometry and its accuracy is estimated to be about ±10 K. It gives about 5%–8% of the error in the retrieved water vapor mixing ratio (Aoki et al., 2019). We also rely on the prediction by GEM‐Mars for the total atmospheric number density and its accuracy is estimated to be about 10%–15% (Aoki et al., 2019). Therefore, it gives another 10%–15% of error in the determined water vapor volume mixing ratio. As for the third source of the additional errors, the most dominant factor is the characterization of the AOTF transfer function. Villanueva et al. (2022) showed that the light from nearby orders reaches detectors through the AOTF. By the solar calibration measurements, these signals from nearby orders are quantified for each diffraction order (Villanueva et al., 2022). The relative amplitude of the signal from nearby orders is smaller in lower diffraction orders, and those in orders 134 and 136 are estimated to be ∼20% (±5%–10%) of the total observed flux (Villanueva et al., 2022). Such signal from nearby orders appears as an offset in the observed spectra. Because the water vapor lines in orders 134 and 136 are usually optically thin, such an offset proportionally impacts the depth of the absorptions, that is, the abundance of water vapor. Given that the uncertainty of the offset is already relatively small (∼5%–10%), the errors in the retrieved water vapor volume mixing ratio are also small (∼5%–10%). Nevertheless, the errors due to uncertainties in GCM and calibration are difficult to quantify. We also perform comparisons of the NOMAD retrievals with the ones retrieved with the simultaneous measurements by ACS to further validate our retrievals (see Section 5).

## 3 Seasonal Variation of Water Vapor Vertical Distributions

**Figure 3:**
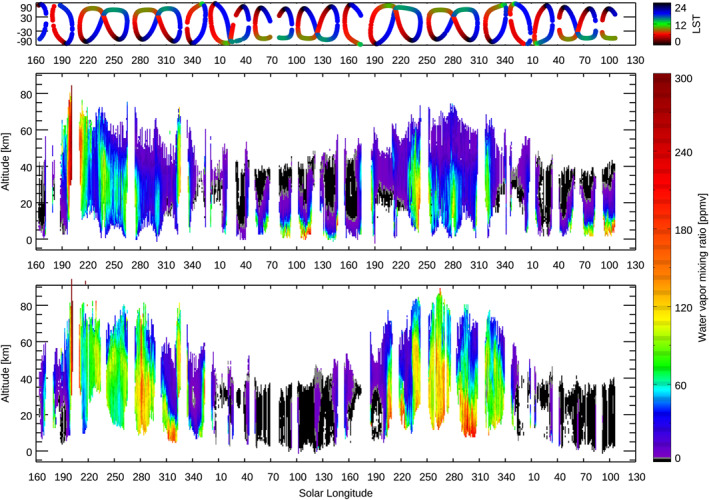
Seasonal variation of the water vapor vertical profiles from *L*_S_ = 160° in MY 34 to *L*_S_ = 130° in MY 36 retrieved from the NOMAD data in the northern hemisphere (the middle panel) and the southern hemisphere (the bottom panel). The retrievals are binned with an interval of 1° of solar longitudes (averaged in latitudes and longitude). The top panel shows the latitudes and local solar time of the measurements (same as Figure 1). The white represents either no detection or no measurement.

Figure 3 shows the seasonal variation of the water vapor vertical profiles retrieved from the NOMAD data set. The retrieved vertical distributions of water vapor exhibit a strong contrast between perihelion and aphelion periods in both hemispheres.

In the northern hemisphere (Figure 3a), the most significant and rapid increase of the water vapor in the middle atmosphere is observed around *L*_S_ = 190° in MY 34. This is driven by the global dust storm that occurred at that period (Aoki et al., 2019). The dust supplied to the atmosphere by the dust storm raises the atmospheric temperature (up to ∼40 K, see Figure 3 of Neary et al., 2020), which prevent water from condensing (Neary et al., 2020). As a result, water vapor in the middle atmosphere is significantly increased and water vapor can reach very high altitude (>∼100 km; Aoki et al., 2019). Another remarkable increase of water vapor in the middle atmosphere appears around *L*_S_ = 320–330° both in MY 34 and MY 35. This is driven by the strong regional dust storm (so‐called “C‐type storm,” Kass et al., 2016) that generally occurs at this moment of the year. Such an increase of the water vapor in the middle atmosphere due to the regional dust storm was found in the previous studies (e.g., Aoki et al., 2019). Chaffin et al. (2021) and Holmes et al. (2021) found that hydrogen escape is also increased after 1 week of the onset of the regional dust storm and argued that the regional dust storm plays an important role in the water escape. This study confirms that the increase of water vapor in the middle atmosphere repeats annually and supports the argument by Chaffin et al. (2021) and Holmes et al. (2021).

In the southern hemisphere (Figure 3b), an increase of water vapor abundances due to the dust storms is also observed. In addition to these, significantly large abundances of water vapor are observed in the southern hemisphere at the local summer periods. Water vapor reaches high altitudes, at least 80 km. Such increases of water vapor around *L*_S_ = 270° have been reported by the recent ACS and NOMAD measurements (Belyaev et al., 2021; Fedorova et al., 2020; Villanueva et al., 2021). This can be explained by the following combination of events occurring at this moment of the year: (a) sublimation of the water vapor from the southern polar ice cap, (b) a strong “one‐cell” meridional circulation from the south to the north, and (c) warmer atmospheric temperatures (i.e., no sequestration of the water vapor by the formation of water ice clouds, see Section 4.2 for more details). Indeed, previous studies with GCMs showed that water vapor indeed can be transported to such high altitudes (Daerden et al., 2019; Holmes et al., 2021; Lefèvre et al., 2004; Shaposhnikov et al., 2019).

**Figure 4:**
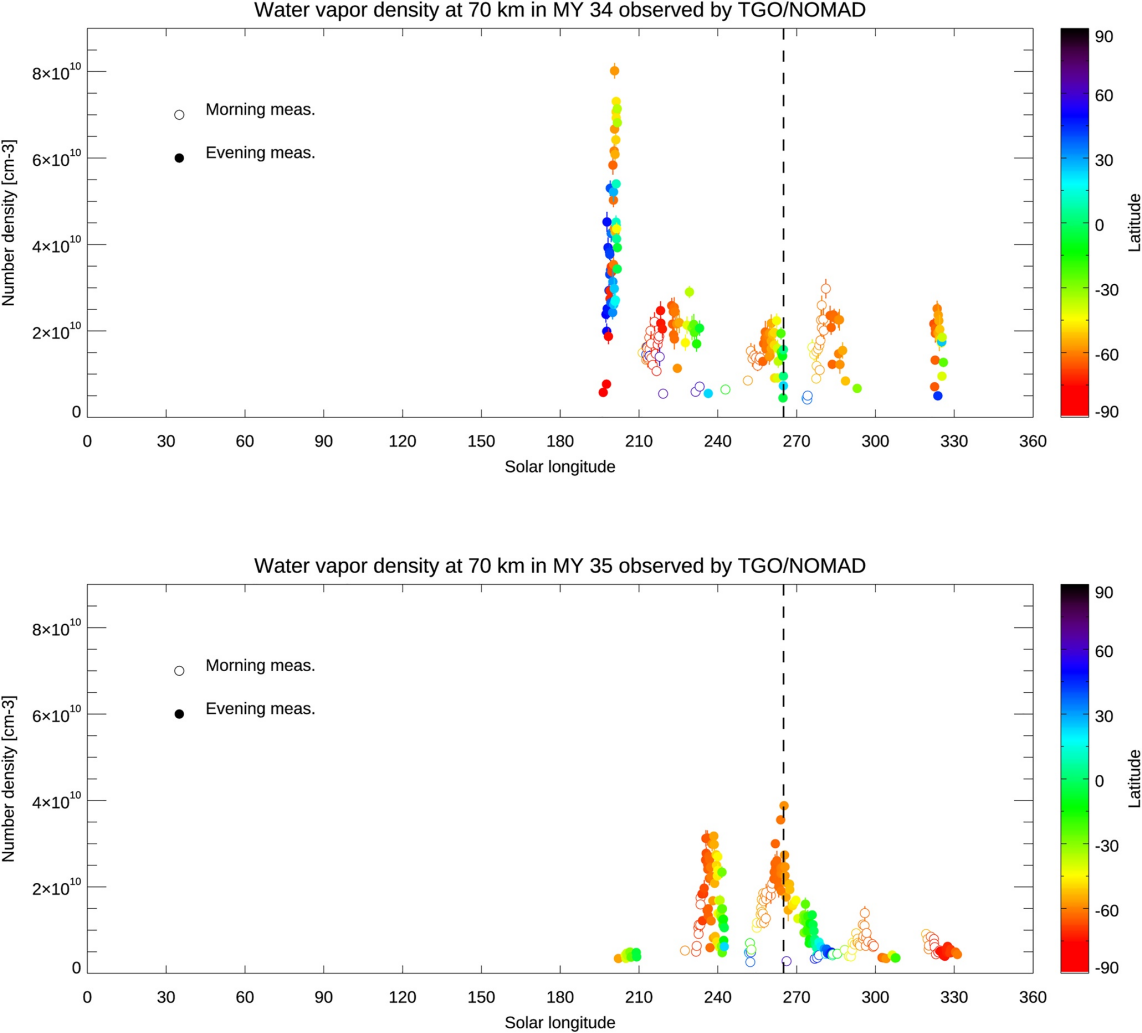
Seasonal variation of the water vapor number density at 70 km altitude in MY 34 (top) and MY 35 (bottom). The differences in color show the latitudes of the measurements. The filled circles represent the measurements in the evening terminator, and the unfilled ones illustrates those in the morning terminator. The retrievals are binned with an interval of 1° of solar longitude (averaged in latitude and longitude). The dashed black lines show the period as *L*_S_ = 265° when the maximum water number density is observed in MY 35.

Figure 4 shows the water vapor number densities at 70 km altitude in MY 34 (Figure 4a) and in MY 35 (Figure 4b). In the first half of Mars years (*L*_S_ = 0–180°), the water vapor number densities are below the detection limit of the NOMAD order 134–136 (∼10^9^ cm^−3^). In contrast, in the latter half of both years (*L*_S_ = 190–330°), water vapor is injected at 70 km following specific events: (a) the global dust storm (*L*_S_ = 190° only in MY 34), (b) southern summer season (*L*_S_ = 240–300° mainly in the southern hemisphere and equator), and (c) regional dust storm in (*L*_S_ = 320–330° in both MY 34 and MY 35). Among these events, the largest water vapor number density at 70 km is observed at the time of global dust storm in MY 34 (reaching ∼8 × 10^10^ cm^−3^), and about half of the highest value is observed in the southern summer period in MY 35 (reaching ∼4 × 10^10^ cm^−3^). In terms of the duration, the water vapor injection due to the *L*_S_ = 190° initiation of MY 34 global dust storm is very large (up to 8 × 10^10^ cm^−3^) but brief, whereas significantly increased number densities are continuously observed in the southern summer season associated with the annual events. In the southern summer season, the largest water vapor number density at 70 km is ∼3 × 10^10^ cm^−3^ at *L*_S_ = 281° in MY 34 and ∼4 × 10^10^ cm^−3^ at *L*_S_ = 265° in MY 35. These maximum values are observed around 60–65°S. The differences of the peak solar longitudes and water vapor number densities between MY 34 and MY 35 during the southern summer season are not argued as interannual variation. This is because the NOMAD measurements at *L*_S_ = 265° in MY 34 (when the maximum water vapor number density is observed in MY 35) do not sample the mid‐southern latitudes (where maximum water vapor number density is observed) such that the differences between MY 34 and 35 are likely a result of differences in the latitudinal sampling. The analysis with ACS data showed a similar variability, which observed the largest water vapor volume mixing ratio at the same period (see Figure 3f of Belyaev et al., 2021). The water vapor number density at the period of the regional dust storm is larger in MY 34, which could be due to the interannual variation as suggested by in Holmes et al. (2021).

**Figure 5:**
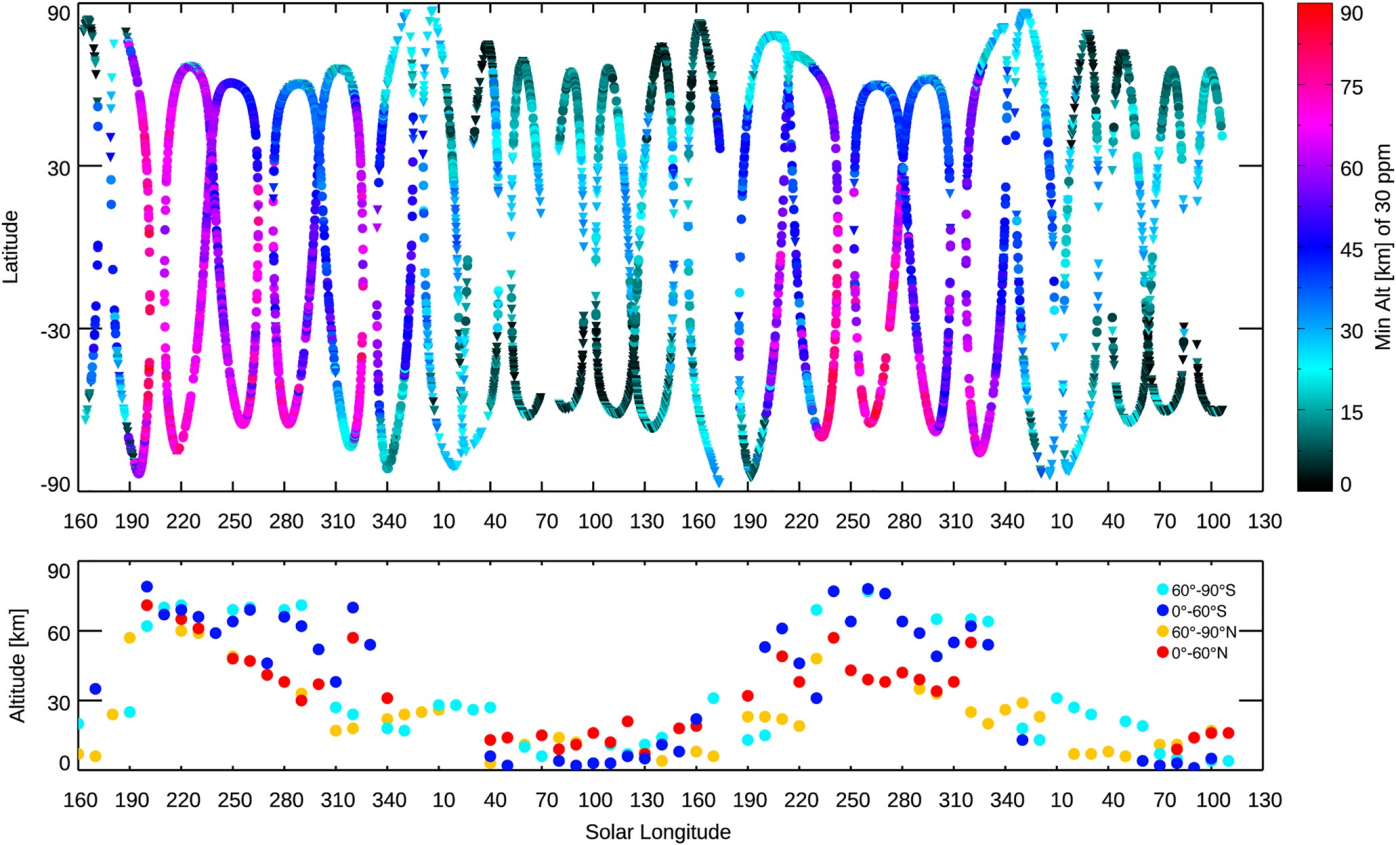
Seasonal and latitudinal variation of the minimum altitudes where the maximum water vapor is less than 30 ppm (the top panel). The bottom panel shows their averaged values with an interval of 10° of the solar longitudes, calculated separately at 90°S‐60°S (light blue), 60°S‐0°S (blue), 0°N‐60°N (red), and 60°N‐90°N (orange).

As described above, the middle atmosphere is generally wet in the perihelion season. In contrast, the middle atmosphere is very dry in the aphelion season. Water vapor sublimated from the northern polar region is confined below 10–40 km, depending on latitude, *L*_S_, and LT over the aphelion season (Figure 3a), and it is severely depleted in the southern hemisphere (Figure 3b). Figure 5 shows the seasonal variation of the maximum altitude where the water vapor volume mixing ratio is below 30 ppm. This figure illustrates well the atmospheric water vapor contrast between aphelion and perihelion—this water vapor confinement level decreases shortly after the end of the annual dust storm activity (around *L*_S_ = 340–350°), reaching a minimum value 10–20 km over *L*_S_ = 40–130°. This confinement level increases to peak altitudes of 50–70 km over *L*_S_ = 190–340°, depending on latitude and dust activity. Large abundances of water can exist in the middle atmosphere (e.g., 40–80 km) in this perihelion period, particularly in the southern hemisphere. This global view is controlled by seasonal change of the atmospheric temperature driven by the Mars‐Sun distance variation and dust loading to the atmosphere, which have been suggested in previous studies (e.g., Clancy et al., 1996; Daerden et al., 2019; Montmessin et al., 2004).

## 4 Latitudinal Variation of Water Vapor Vertical Distributions

**Figure 6:**
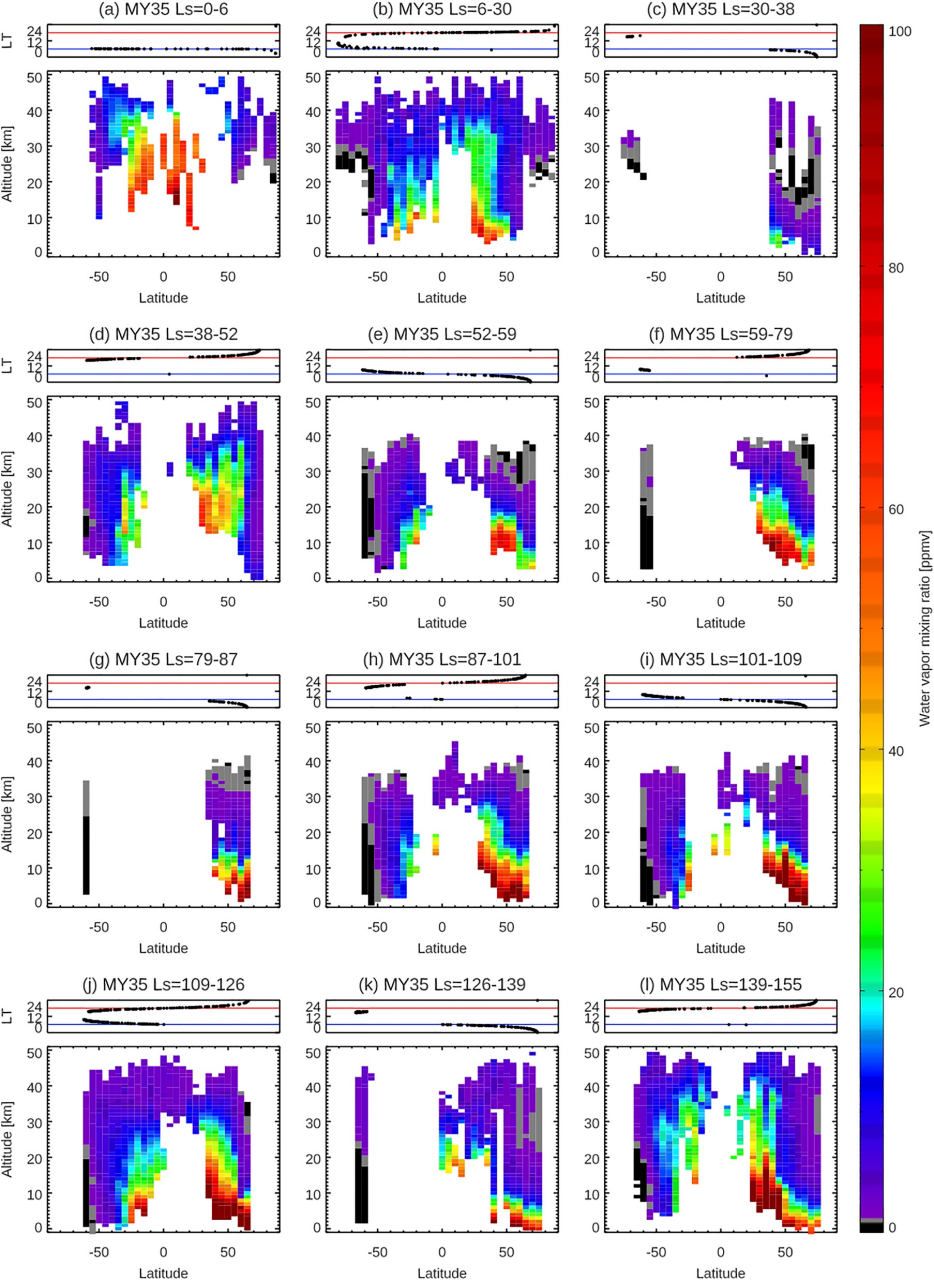
Latitudinal variation of the water vapor vertical profiles retrieved from Nadir and Occultation for Mars Discovery (NOMAD) data at *L*_S_ = 0–6° (a), *L*_S_ = 6–30° (b), *L*_S_ = 30–38° (c), *L*_S_ = 38–52° (d), *L*_S_ = 52–59°(e), *L*_S_ = 59–79° (f), *L*_S_ = 79–87° (g), *L*_S_ = 87–101° (h), *L*_S_ = 101–109° (i), *L*_S_ = 109–126° (j), *L*_S_ = 126–139° (k), and *L*_S_ = 39–155° (l) in MY 35. The retrievals are binned with an interval of 5° of latitudes (averaged in longitude). The top panels show the local times of the measurements for each figure. The blue and red lines illustrate 6 a.m. and 6 p.m., respectively.

**Figure 7:**
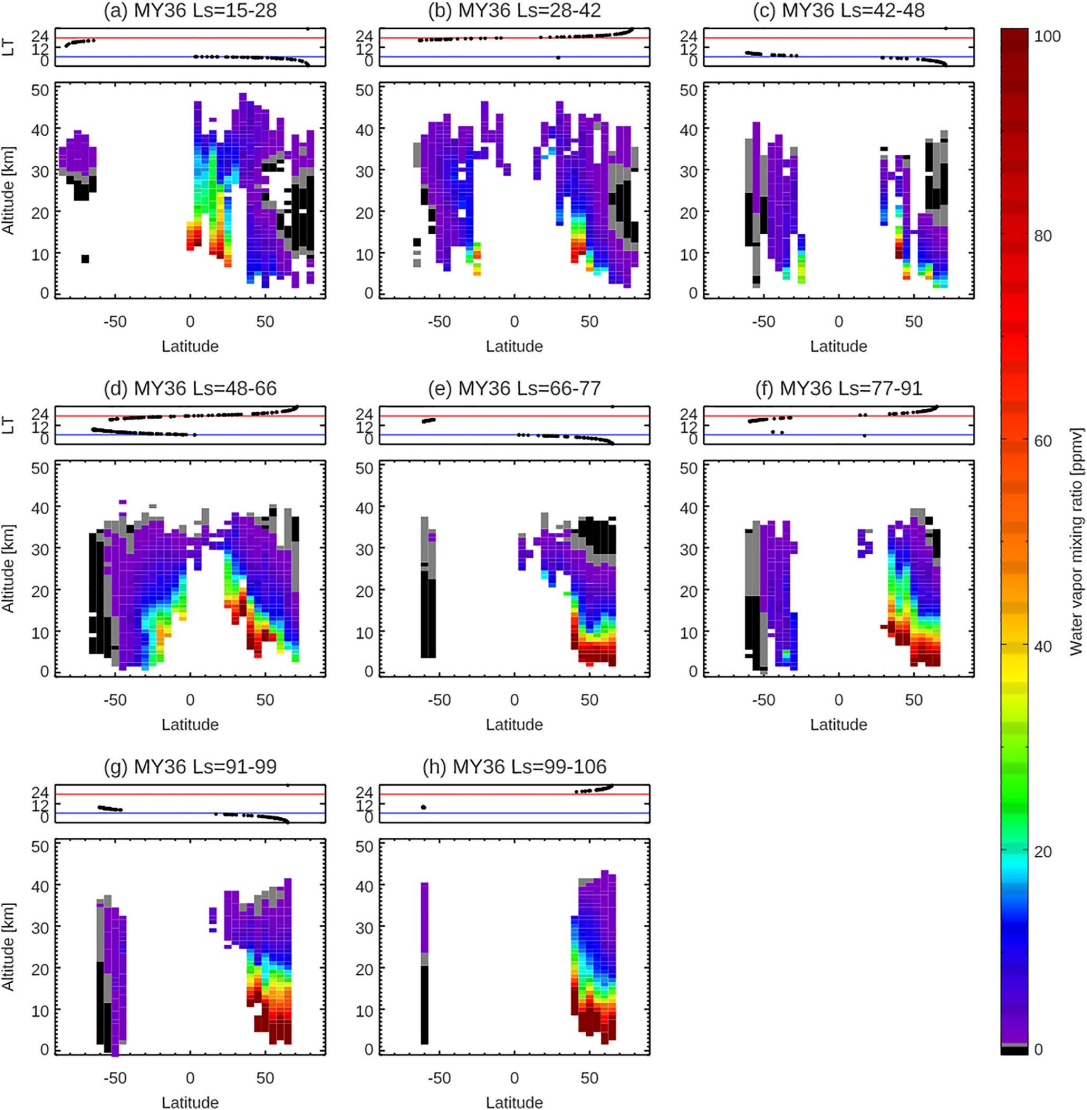
Latitudinal variation of the water vapor vertical profiles retrieved from Nadir and Occultation for Mars Discovery (NOMAD) data at *L*_S_ = 15–28° (a), *L*_S_ = 28–42° (b), *L*_S_ = 42–48° (c), *L*_S_ = 48–66° (d), *L*_S_ = 66–77°(e), *L*_S_ = 77–91° (f), *L*_S_ = 91–99° (g), and *L*_S_ = 99–106° (h) in MY 36. The retrievals are binned with an interval of 5° of latitudes (averaged in longitude). The top panels show the local times of the measurements for each figure. The blue and red lines illustrate 6 a.m. and 6 p.m., respectively.

**Figure 8:**
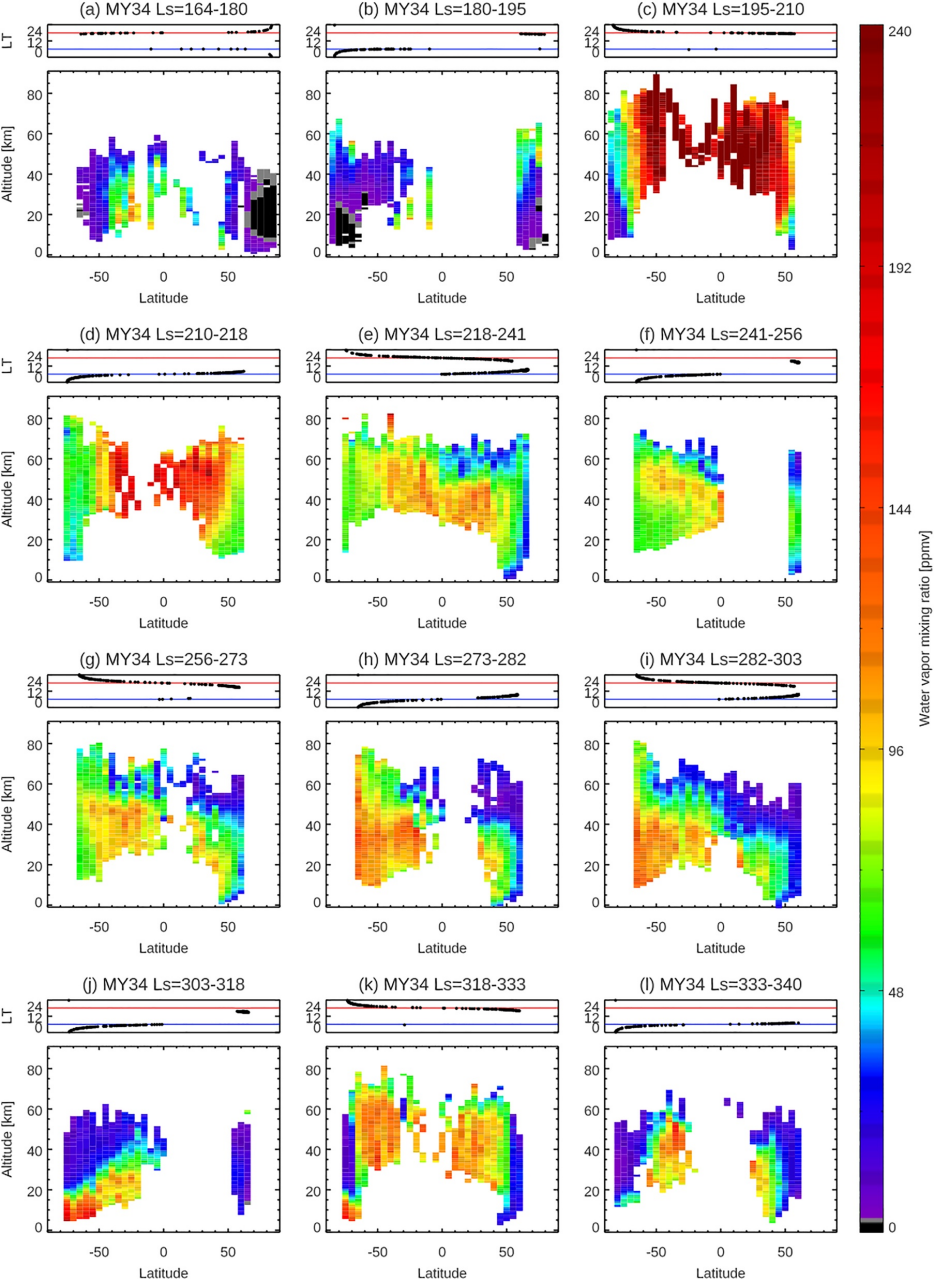
Latitudinal variation of the water vapor vertical profiles retrieved from Nadir and Occultation for Mars Discovery (NOMAD) data at *L*_S_ = 164–180° (a), *L*_S_ = 180–195° (b), *L*_S_ = 195–210° (c), *L*_S_ = 210–218° (d), *L*_S_ = 218–241°(e), *L*_S_ = 241–256° (f), *L*_S_ = 256–273° (g), *L*_S_ = 273–282° (h), *L*_S_ = 282–303° (i), *L*_S_ = 303–318° (j), *L*_S_ = 318–333° (k), and *L*_S_ = 330–340° (l) in MY 34. The retrievals are binned with an interval of 5° of latitudes (averaged in longitude). The top panels show the local times of the measurements for each figure. The blue and red lines illustrate 6 a.m. and 6 p.m., respectively.

**Figure 9:**
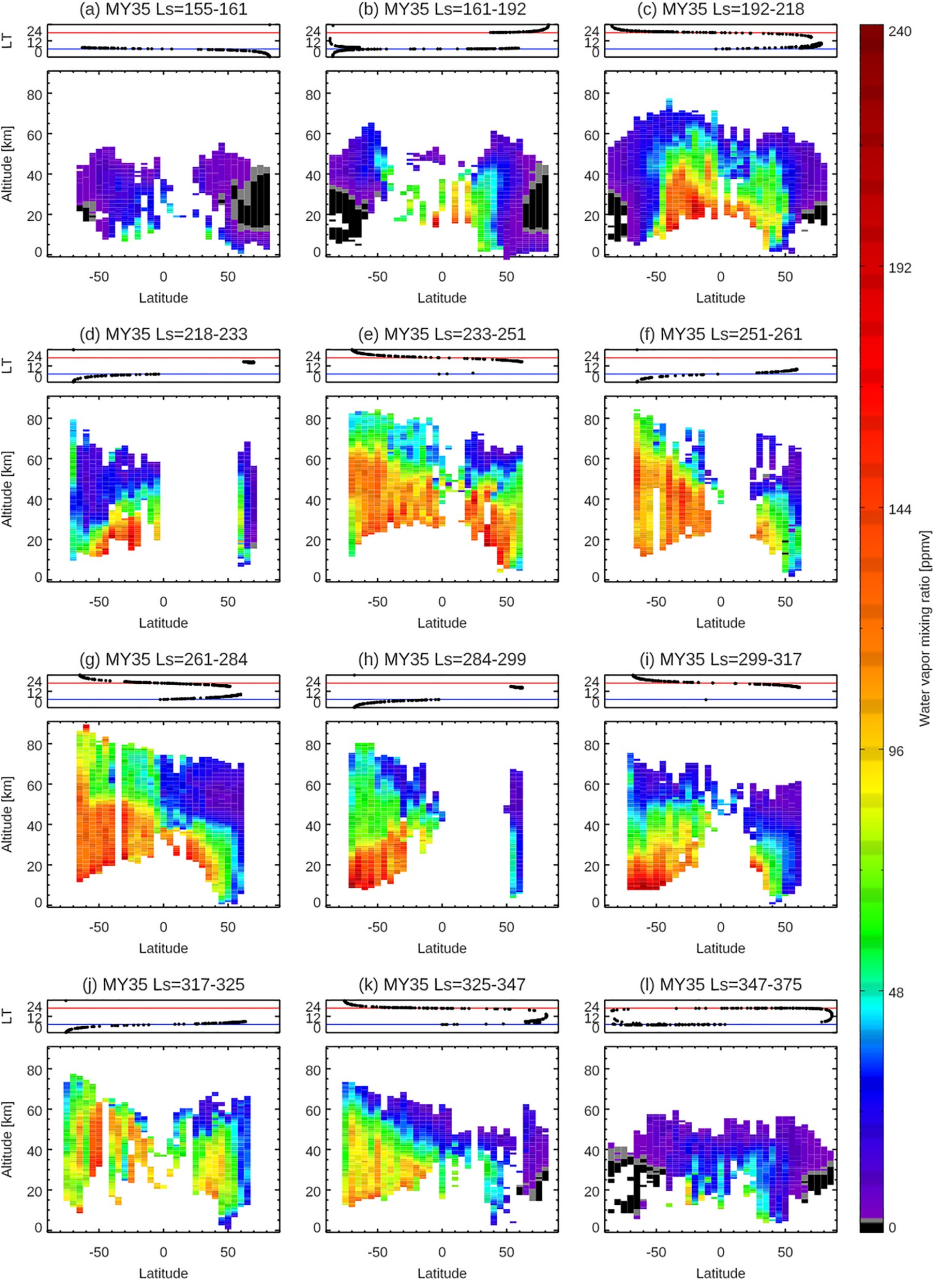
Latitudinal variation of the water vapor vertical profiles retrieved from Nadir and Occultation for Mars Discovery data at *L*_S_ = 155–161° (a), *L*_S_ = 161–192° (b), *L*_S_ = 192–218° (c), *L*_S_ = 218–233° (d), *L*_S_ = 233–251°(e), *L*_S_ = 251–261° (f), *L*_S_ = 261–284° (g), *L*_S_ = 284–299° (h), *L*_S_ = 299–317° (i), *L*_S_ = 317–325° (j), *L*_S_ = 325–347° (k) in MY 35, and from *L*_S_ = 347° in MY 35 to *L*_S_ = 15° in MY 36 (l). The retrievals are binned with an interval of 5° of latitudes (averaged in longitude). The top panels show the local times of the measurements for each figure. The blue and red lines illustrate 6 a.m. and 6 p.m., respectively.

The large‐scale seasonal variation of Mars water vapor vertical distributions is visually captured in Figure 3. However, the detailed seasonal, latitudinal, and LT variations are not separated in these presentations and so are less apparent. In this section, to distinguish among them, a series of latitude–altitude maps of water vapor within seasonal bins are visualized. Figures 6–9 show the latitude‐altitude maps of the zonal averaged water vapor volume mixing ratio in the aphelion periods of MY 35 (Figure 6) and MY 36 (Figure 7), and in perihelion periods of MY 34 (Figure 8), and MY 35 (Figure 9). The sampling local times are shown at the top panels of each figure. The irregular seasonal intervals in these figures are decided based on the moment when local time of the NOMAD measurements switch between morning (6a.m. ± 6 hr) and evening (6p.m. ± 6 hr), which corresponds to the moment when the NOMAD measures are performed at the highest latitudes. This way, we can distinguish variability in latitude, local solar time, and season as much as possible. Note that we use the areoid altitudes in all of the figures.

**Figure 10:**
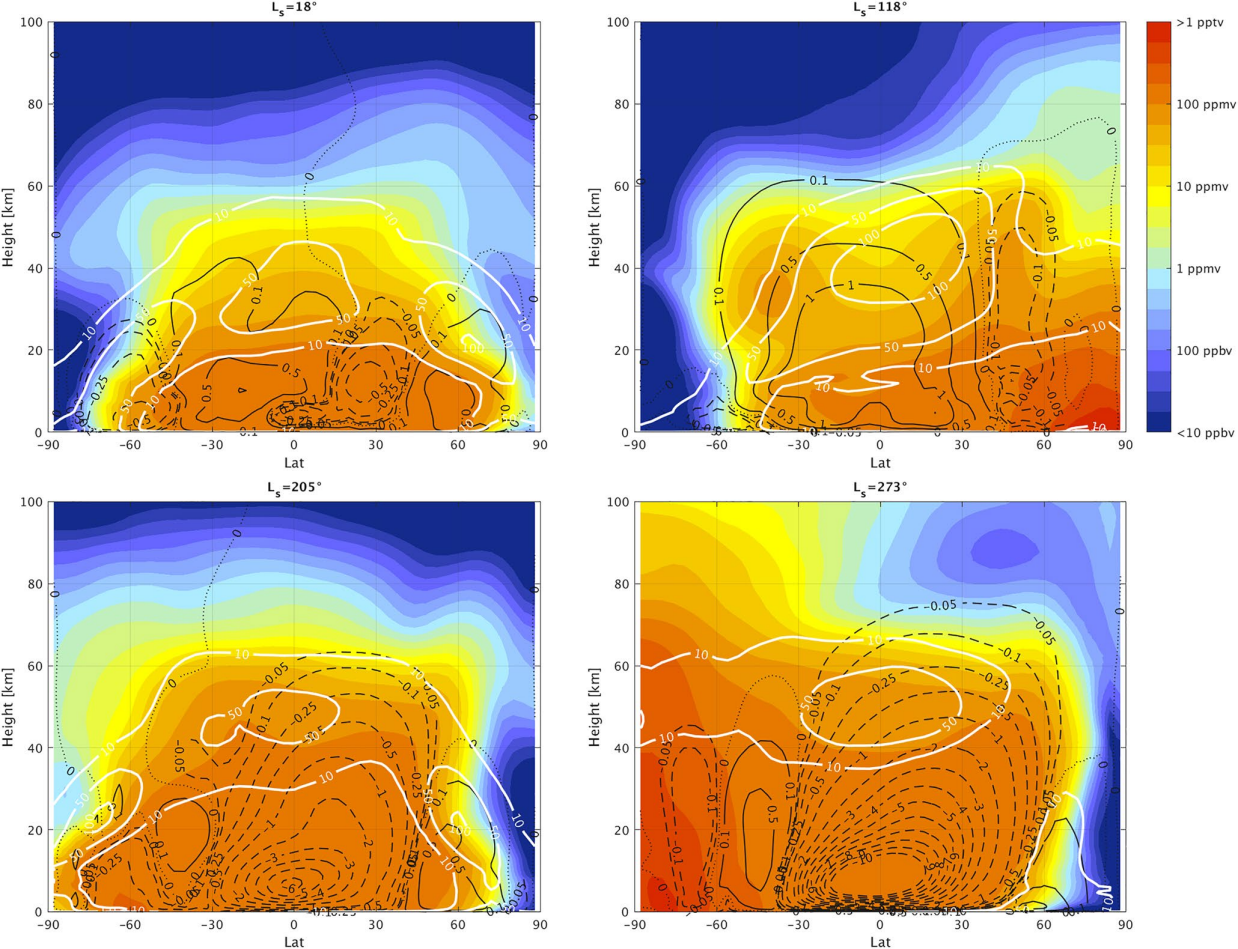
Latitude—altitude maps of water vapor vertical distributions calculated by GEM‐Mars (Daerden et al., 2019, 2022; Neary et al., 2020) at *L*_S_ = 18° (a), *L*_S_ = 118° (b), *L*_S_ = 205° (c), and *L*_S_ = 273° (d) for non‐global dust storm year such as MY 35. The seasons are selected to represent the Nadir and Occultation for Mars Discovery results (Figures 7a, 7e and 10b, 10d) The color shading denotes the volume mixing ratio of water vapor. The white and black contours illustrate the volume mixing ratio of water in ice clouds (ppmv, see text) and mass stream functions (×10^9^ kg/s), respectively. Full lines represent counterclockwise movement, and dotted lines represent clockwise movement of air. The values are averaged over 5 sols.

**Figure 11:**
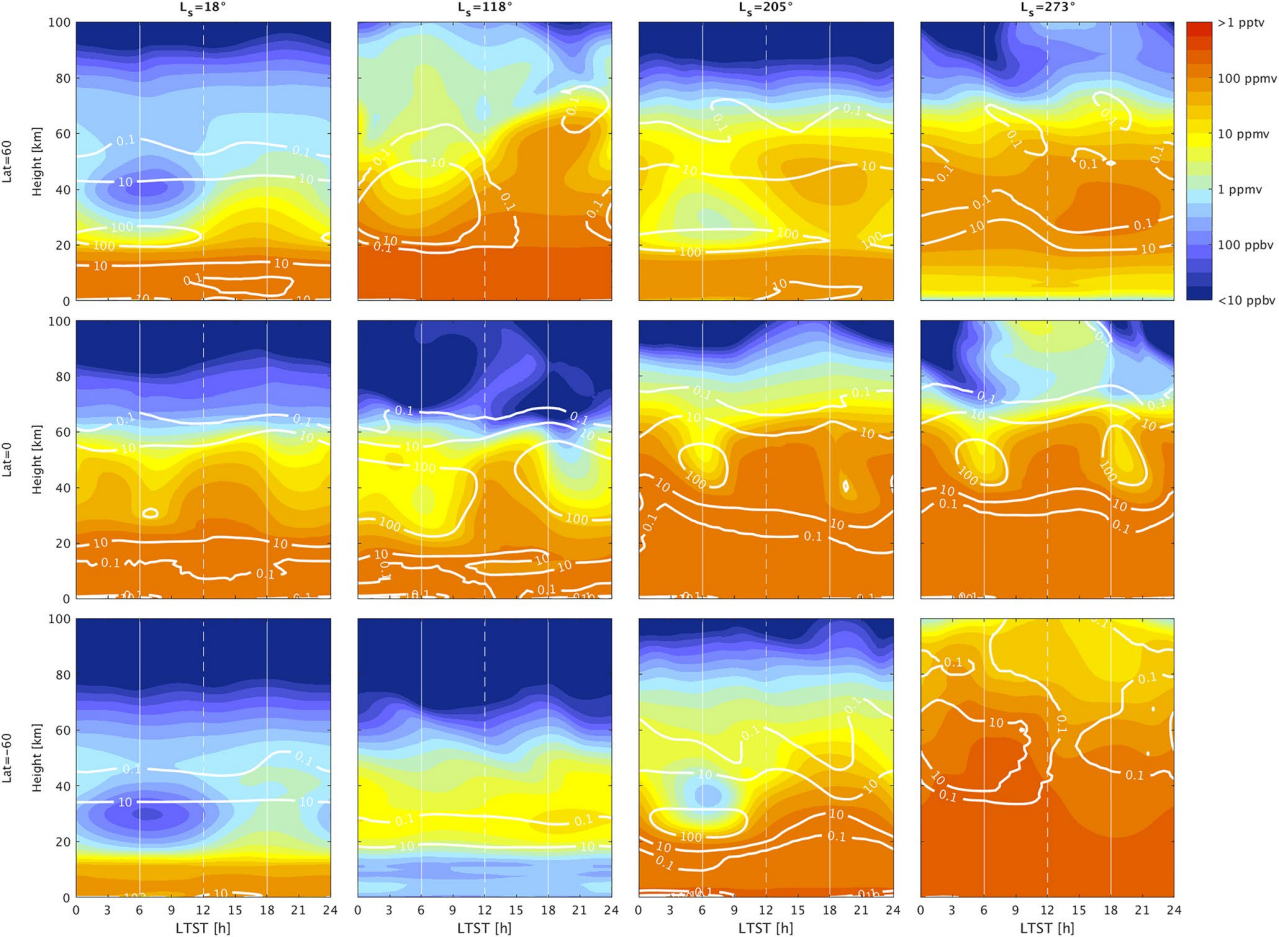
Local time variation of the water vapor vertical distributions calculated by GEM‐Mars (Daerden et al., 2019, 2022; Neary et al., 2020) at *L*_S_ = 18° (a), *L*_S_ = 118° (b), *L*_S_ = 205° (c), and *L*_S_ = 273° (d) for non‐global dust storm year such as MY 35. The seasons are selected to represent the Nadir and Occultation for Mars Discovery results (Figures 7a, 7e and 10b, 10d) The color shading denotes the volume mixing ratio of water vapor and the white contours illustrate the volume mixing ratio of water in ice clouds (ppmv). The top, middle, and bottom panels show results at 60°N, 0°N, and 60°S, respectively. The values are averaged over 5 sols.

The latitude‐altitude distributions and local time variations can be interpreted using the simulations of the water vapor distributions in the GEM‐Mars GCM (Daerden et al., 2019, 2022; Neary et al., 2020). Figure 10 show the latitude‐altitude maps of the water vapor vertical distributions simulated by GEM‐Mars, averaged over 5 sols at representative seasons of the NOMAD measurements (L_S_ = 18°, 118°, 205°, and 273° for non‐global dust storm year such as MY35). Figure 11 shows the simulated LT variation of the water vapor profiles at 3 selected latitudes (60°N/S and equator), also averaged over 5 sols at the same seasons. These figures also contain simulated water ice clouds mixing ratio (defined as number density of condensed water relative to air density) and mass stream function (at Figure 10). Generally, observed distributions of water vapor follow the meridional circulations as predicted by the GCM.

### 4.1 Northern Spring‐Summer Season

Shortly after the *L*_S_ = 0° northern vernal equinox, most of water vapor is confined between 40°S and 60°N, extending to ∼30–40 km altitudes (optimal latitude coverage in MY 35 (Figures 6a–6d), but also represented in MY 36 (Figures 7a–7d)). The peak abundance and vertical extent of water vapor is greater in the northern hemisphere, with maximum values around 30°N in the earlier period (*L*_S_ = 6–30°, Figure 6b) and an increase is observed at 40–60°N in the later period (*L*_S_ = 38–52° in Figure 6d, *L*_S_ = 48–66° in Figure 7d). Higher latitudes (poleward of 60° both in the north and south) are very dry at all altitudes in this early northern spring period. The decrease of water vapor around 30–40 km at this time is likely associated with the formation of water ice clouds, which is detected every Mars year by Mars Climate Sounder (MCS) onboard Mars Reconnaissance Orbiter (MRO) (e.g., Guha et al., 2021; McCleese et al., 2010), observed by TGO/NOMAD in MY 35 (Stolzenbach et al., 2022), and simulated in GEM‐Mars (Figure 10a). The observed vertical distribution of water vapor is consistent with the meridional circulation in the northern vernal equinox with broad upwelling at low‐middle latitudes suggested by MCS measurements (e.g., Heavens et al., 2011) and GEM‐Mars (Figure 10a). This period reflects the transition from equinoctial high ice cloud distributions to the early formation of the lower altitude/latitude aphelion cloud belt (see Figures 16, 17a, 17b, and 17c in McCleese et al., 2010). The observed water vapor distribution is asymmetric about the equator, in that it tends to be more pronounced in the northern hemisphere.

**Figure 12:**
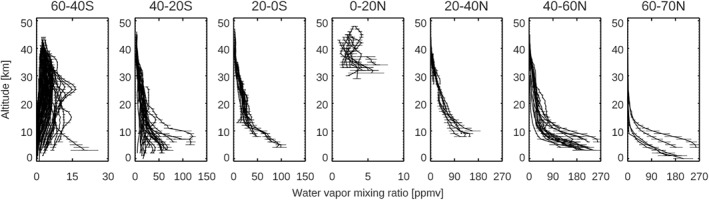
Vertical profiles of the retrieved water vapor volume mixing ratio at *L*_S_ = 109–126° in MY 35, which corresponds to the map shown in Figure 6j. The profiles are illustrated in separate panels based on the latitudes of the measurements (from the left to the right: Lat = 60‐40°S, 40‐20°S, 20‐0°S, 0–20°N, 20–40°N, 40–60°N, 60–70°N). Note that the horizontal scales are different at each panel.

The water vapor maps around the northern summer solstice (e.g., *L*_S_ = 80–140°) in MY 35 and MY 36 are shown in Figures 6g–6k and 7f–7h, respectively. The individual vertical profiles of the retrieved water vapor volume mixing ratio for *L*_S_ = 109–126° (Figure 6j) are also shown in Figure 12. In the northern summer solstice, significantly large abundances of water vapor are observed in the northern high latitudes (>50–60°N) due to the sublimation of the northern polar ice, with a maximum volume mixing ratio of ∼240 ppm around 3 km above areoid (the lower altitude limit of the NOMAD retrievals in this period) at *L*_S_ = 109–126° (Figures 6j and 12). These large abundances of water vapor at the northern high latitudes decrease rapidly with altitude, with an order of magnitude in mixing ratio by 15 km altitude. Water vapor is notably less confined at the northern low‐middle latitudes (below ∼40°N), with an order of magnitude reduction in mixing ratio by ∼30 km altitudes. The southern hemisphere greater than 40°S is very dry in the whole altitude range, however, significant amounts of water vapor (20–80 ppm, Figure 12) are present at the low latitudes (0–40°S) mostly below ∼10–20 km. The vertical extent of water vapor over the equatorial ranges gradually increases with season (reaching ∼40–50 km around *L*_S_ = 140–150°, Figure 6l).

Confinement of water vapor at the nighttime, northern subpolar latitudes (>∼40°N) observed by NOMAD in the northern summer solstice is most likely caused by low‐altitude ice clouds, such as observed by the lidar on the Phoenix mission (68°N) in this season (Whiteway et al., 2009). Model interpretation of the Phoenix ice cloud observations demonstrated that large ice particles cause precipitation (consistent with the fall streaks observed by the lidar) and thus confine the water vapor confined to low altitudes (Daerden et al., 2010). The predicted meridional circulation in the northern summer solstice is characterized by a large Hadley cell from ∼40°N to ∼40°S with the presence of small cells isolated at the polar regions (Figure 10b). This suggests that once the water vapor released from the polar cap reaches the upwelling branch of the main meridional circulation around 40°N, water vapor is transported vertically and towards the southern hemisphere, although significantly constrained by ice formation and gravitational confinement in the lower latitude aphelion cloud belt. The GEM‐Mars model simulates such a thick layer of water ice clouds around 20–30 km over the northern tropics at the cold atmospheric layer at the upwelling branches of the main meridional cell (Figure 10b), which was also reported by the MCS measurements (Heavens et al., 2011; McCleese et al., 2010). It was proposed that the cold layer prevents water from transporting from the northern to the south hemisphere by forming water ice clouds (so‐called “Clancy effect,” Clancy et al., 1996). The NOMAD measurements show the presence of water vapor over the equatorial regions in the southern hemisphere (0–30°S) mostly below ∼20 km. The aphelion cloud belt limits the vertical extent of the water vapor and reduces the water vapor transport by meridional circulation, however, water vapor is still transported from the northern hemisphere to the southern hemisphere. This is consistent with the GCM simulation by Kahre et al. (2020) suggested that the north‐south water transport at this season is not done by the meridional circulation but stationary eddies. The GEM‐Mars model over‐predicts the water vapor abundances at high northern latitudes (Figure 10b), which is due to the simple water ice cloud representation (Daerden et al., 2022), which does not confine water vapor sufficiently at high northern latitudes when it sublimates from the cap.

Comparison between northern middle latitudes (30–50°N) water vapor measurements in the morning terminator (Figures 6b–6f, 6h, 6j, 6l and 7b–7f, 7h) and the evening terminator (Figures 6a–6e, 6i, 6k and 7a–7e) suggests that the water vapor is more vertically extended in the evening terminator than in the morning terminator. This may be due to the local time variation of the water ice clouds formation driven by the diurnal cooling (e.g., Wilson & Richardson, 2000). GEM‐Mars simulations show that the diurnal variation of water vapor is associated with the formation of water ice clouds (Figure 11b). Less cloud formation in the daytime prevents water vapor from remaining confined and thus permits more vertical transport of water.

The morphology of the water vapor distributions in the first half of MY 36 shown in Figure 7 is quite similar to those in MY 35 (Figure 6), which confirms that the features discussed above are annually repeated.

### 4.2 Southern Summer‐Spring Season

Water vapor vertical distributions near and shortly after the northern autumnal equinox (Figures 9a–9c) are somewhat similar to those during/shortly after the northern vernal equinox (Figures 6a and 6b): water vapor is confined into low‐middle latitudes (within about ±50°) and high latitudes present very dry atmospheres. However, compared to the northern vernal equinox, water vapor abundances and vertical extents in the middle atmosphere are larger in the case of the northern autumnal equinox—the maximum volume mixing ratio and the vertical extent of water vapor observed by NOMAD reach ∼210 ppm and up to ∼40–50 km, respectively. This is presumably related to the higher altitudes of the water ice clouds formation, which is suggested in GEM‐Mars model at the northern autumnal equinox (∼40–50 km, Figure 10c). The observed water vapor vertical distribution is also asymmetric about the equator, in that it is more pronounced in the southern hemisphere (Figure 9c).

Around the northern winter/southern summer solstice (e.g., *L*_S_ = 230–280° in Figures 8g and 8h and 9e–9g), large amounts of water vapor (>100 ppm) are widely present in the middle atmosphere except at northern high latitudes. In particular, water vapor reaches very high altitudes (at least 80 km) at southern high latitudes (greater than 50°S). This distribution can be explained as follows: water vapor sublimated from the southern polar ice cap is directly captured by the more intense perihelion meridional circulation (from the south to the north). Note that Figure 10 shows the diurnally averaged mass stream function, but at daytime a strong Hadley cell develops in the southern hemisphere that extends to above 60 km (not shown). The global atmosphere is warm enough to suppress water ice clouds because of the presence of the dust in the atmosphere and shorter Sun‐Mars distance (i.e., increased solar heating). Thus, the water vapor captured by the strong upward branches of the daytime Hadley cell is effectively transported to the upper atmosphere, and by a pole‐to‐pole meridional cell towards the northern hemisphere. This behavior is well matched by GCM simulations (e.g., Lefèvre et al., 2004; Daerden et al., 2019; Shaposhnikov et al., 2019, also Figure 10d). The maximum abundance of water vapor (∼240 ppm) is observed over the southern high latitudes at *L*_S_ = 284° but the abundance of water vapor in the middle atmosphere decreases gradually after *L*_S_ = 284° (Figures 8j and 9h–9i). However, it rapidly increases again widely around *L*_S_ = 320°, at the time of the annual strong regional dust storm (Figures 8k and 9j). The enhanced water vapor widely present over the middle atmosphere at the southern summer solstice (Figures 8l and 9k) rapidly decreases after *L*_S_ = 347° (Figure 9l). This is presumably due to the fact that the global atmosphere is cooling, and the supply of perihelion water vapor from the receding southern residual cap no longer exists once only the residual CO_2_ ice cap is present over the southern polar region.

The observed seasonal variation of the latitude‐altitude maps of water vapor in the latter half of MY 35 is consistent with the results by TGO/ACS (Belyaev et al., 2021). The latitude‐altitude maps of the zonal averaged water vapor volume mixing ratio in the latter half of the MY 34 shown in Figure 8 are consistent with the results by ACS (Fedorova et al., 2020) and previous studies with the NOMAD data (Aoki et al., 2019; Villanueva et al., 2021). The morphologies of the latitude‐altitude maps of water vapor in MY 34 are similar to those in MY 35, apart from the period of the global dust storm in MY 34.

Figures 8 and 9 show that the difference of the vertical distribution of water vapor between morning and evening terminator in the perihelion portion of the Mars years is less obvious. This provides additional evidence that the formation of the water ice clouds, which drives morning‐evening differences in the first half of Mars year, does not play an important role in the latter half of Mars years. This is confirmed by the simulations of GEM‐Mars (Figure 11d).

## 5 Comparison With ACS NIR

**Figure 13:**
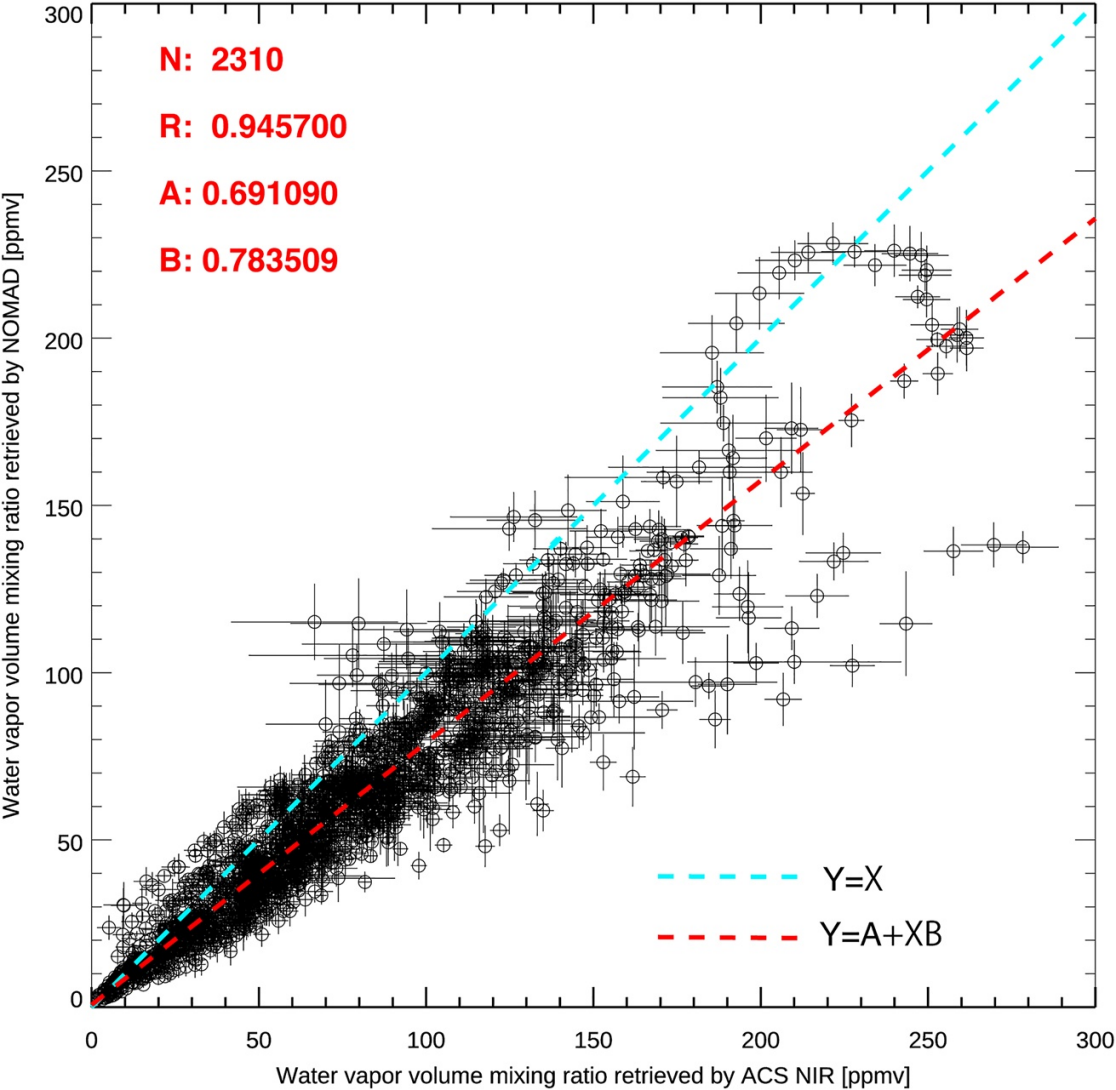
Comparison of the water vapor volume mixing ratio retrieved by Atmospheric Chemistry Suite (ACS) NIR (*x*‐axis) and Nadir and Occultation for Mars Discovery (*y*‐axis) using their simultaneous measurements. The water vapor volume mixing retrieved by ACS NIR is recalculated based on the atmospheric density predicted by GEM‐Mars that are used to calculate the water vapor volume mixing ratio retrieved by Nadir and Occultation for Mars Discovery (NOMAD). The light‐blue and red and lines show line of equality and best‐fit linear functions, respectively. N, R, A, B values represent the number of the samples, correlation factor (the linear Pearson correlation coefficient), and the coefficients of the best‐fit linear function (NOMAD_vmr_ = *A* + ACS_vmr_ × *B*, where NOMAD_vmr_ and ACS_vmr_ stand for the water vapor volume mixing ratio retrieved by NOMAD and ACS NIR).

ACS is another instrument onboard TGO, which is able to perform spectroscopic measurements of water vapor using the solar occultation technique (Alday et al., 2021; Belyaev et al., 2021; Fedorova et al., 2020). ACS has two channels in the near infrared range: one is the near‐infrared (NIR) channel working at shorter wavelength (0.73–1.6 μm) based on the AOTF filter and echelle‐grating, and the other one is the mid‐infrared (MIR) channel working at longer wavelength (2.3–4.3 μm) based on echelle diffraction grating and secondary cross‐dispersion diffraction gratings (Korablev et al., 2018). Solar occultation measurements by NOMAD and ACS NIR are co‐aligned, thus simultaneous observations are possible. We have performed about 2,000 simultaneous solar occultation measurements between NOMAD and ACS NIR for the 3.5 years of the TGO science operation. In this study, we compare the water vapor retrievals from NOMAD and ACS NIR using the simultaneous measurements taken between July 2018 and March 2019, corresponding to *L*_S_ = 218–353° in MY 34. This includes the periods of the global and regional dust storms. In total, 2307 retrieved water vapor abundances in 77 occultations are available for the comparison in this period. We have used the most recent version of the ACS NIR retrievals (Fedova et al., 2022). The procedure of the ACS NIR retrievals can be found in Fedorova et al. (2020). In the retrievals of ACS NIR data, spectra at all altitudes are processed all at once, which is different from the method of our analysis that processes spectra at each altitude independently. Figure 13 shows the comparison between the water vapor volume mixing ratios retrieved from ACS NIR (*x*‐axis) and NOMAD (*y*‐axis). To eliminate the effect that we use different atmospheric densities to calculate the water vapor volume mixing ratio, the ACS NIR retrievals were calculated by the ratio between water vapor number densities retrieved from the ACS NIR data and the atmospheric densities predicted by GEM‐Mars (which was used to calculate NOMAD retrievals). The vertical structures of the water vapor volume mixing ratio are quite similar (not shown). The correlation factor is 0.95, which means that NOMAD and ACS NIR retrievals are highly linearly correlated. The best‐fit linear function of the comparison is NOMAD_vmr_ = 0.69 + ACS_vm*r*_ × 0.78, where NOMAD_vmr_ and ACS_vmr_ stand for the water vapor volume mixing ratio retrieved by NOMAD and ACS NIR, respectively. It suggests that NOMAD water vapor density retrievals are 22% systematically smaller than ACS NIR density retrievals. Given that this is a comparison between results derived from different instruments, spectral ranges, and methodologies, the agreement is good and within the range of uncertainty. NOMAD and ACS NIR retrievals are obtained with different methodologies and spectral intervals, which are likely the primal sources of the discrepancy. This aspect will be investigated further in future.

## 6 Saturation Ratio of Water Vapor

How much water vapor can be present in excess of saturation (i.e., super‐saturation, as expressed by the saturation ratio) is an important parameter to quantify the amount of water that can be transported to the upper atmosphere. Hence, potential levels of super‐saturation bear on atmospheric water escape, which affects the long‐term atmospheric evolution of the terrestrial planets. In the atmosphere of the Earth, super‐saturation is common in the upper troposphere where saturation ratio is ∼1.5 (Gettelman et al., 2006). The occurrences of super‐saturation have been also reported in the atmosphere of Mars, however involving much higher values. Maltagliati et al. (2011) showed that super‐saturation could be frequently present in the northern spring‐summer season in around 30–50 km both in the northern and southern hemisphere and the saturation ratio reaches about 10. They used GCM temperatures to derive these values, which will contribute to significant uncertainties, particularly in combination with the extensive atmospheric path lengths associated with limb viewing geometry. Poncin et al. (2022) showed that super‐saturation conditions are rare for the Mars atmosphere below ∼50 km, based on water abundances estimated from the measurements by Compact Reconnaissance Imaging Spectral Mapper (CRISM) onboard MRO and co‐located temperature retrieved from the MRO/MCS measurements. Fedorova et al. (2020) showed that a super‐saturation occurs at 80–100 km altitude during the northern autumn‐winter season and the saturation ratio sometimes reaches above 100 (Figure 2 of Fedorova et al., 2020), based on water vapor and temperature retrieved from the ACS NIR data.

**Figure 14:**
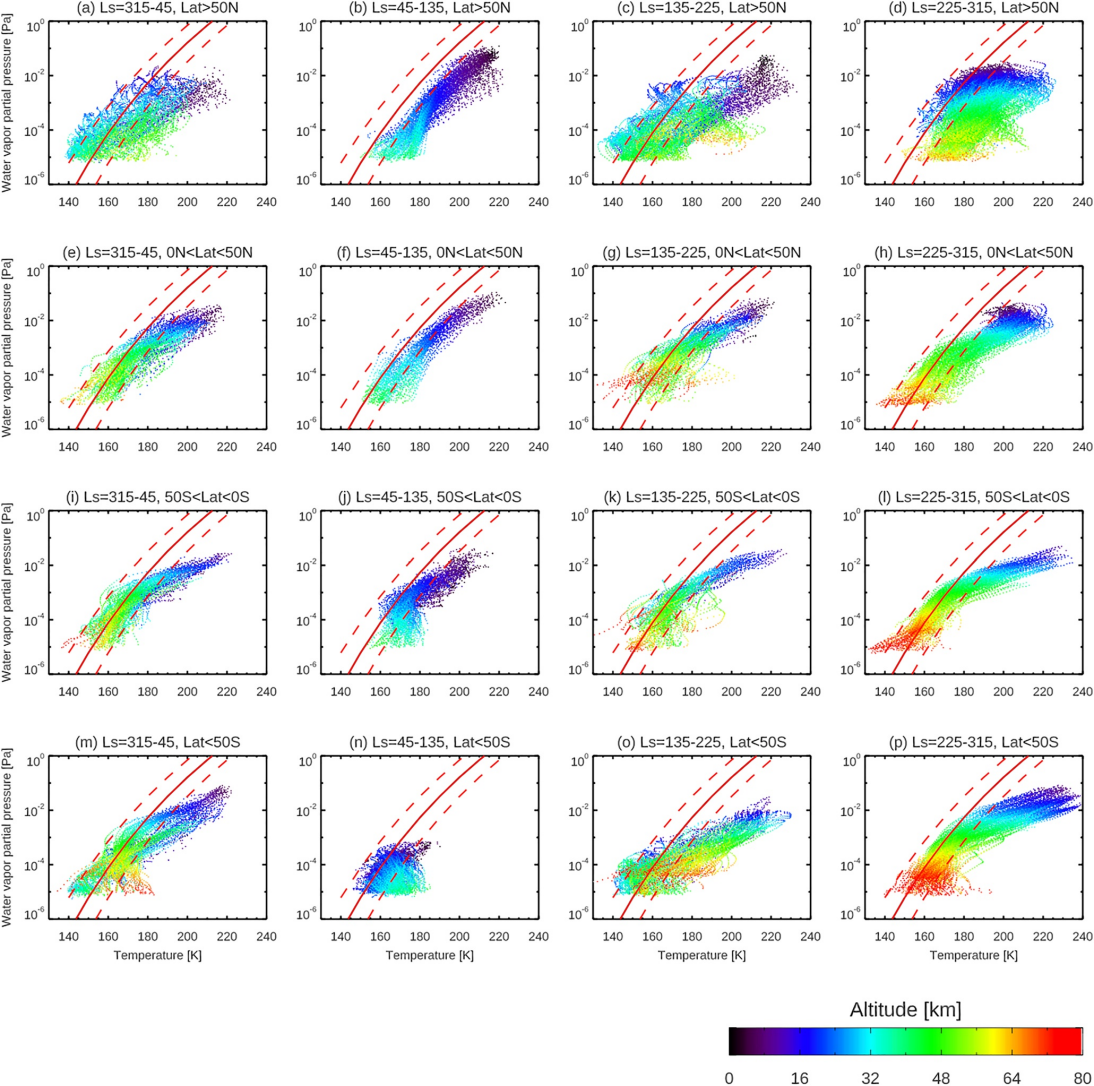
Water vapor partial pressures determined in this analysis. The differences in colors represents the altitude of the measurements. They are illustrated in the separate panels depending of the seasons and latitude of the measurements. The red thick curves show the saturation vapor pressure, and the red dashed curves are the range of their accuracy due to the uncertainty in the general circulation model (GCM) temperatures (±10 K).

In this study, we do not yet have observed temperatures from the NOMAD measurements corresponding to the altitude range investigated here, although temperatures from CO_2_ density measurements by NOMAD have been recently processed (Trompet et al., 2022). We therefore relied on temperatures predicted by the GEM‐Mars (Daerden et al., 2019; Neary et al., 2020). As mentioned above, this introduces uncertainties. Nevertheless, it is instructive to see the results of the magnitude of super‐saturation and the locations and seasons of super‐saturation conditions based on the NOMAD water vapor retrievals. Figure 14 shows the water vapor partial pressures determined by this analysis and the saturation vapor pressure calculated by Equation 7 in Murphy and Koop et al. (2005) (the thick red curves in Figure 14). They are illustrated in the separate panels depending on the seasonal and latitudinal ranges (*L*_S_ = 315‐45°, 45–135°, 135–225°, 225–315°, Lat = 90‐50°S, 50‐0°S, 0–50°N, 50–90°N). As described in Section 2, we estimate the accuracy of temperature predicted by GEM‐Mars is about ±10 K, which introduces large uncertainties in the saturation vapor pressure (the dashed red curves in Figure 14), as expected. Figure 14 demonstrates that such a large uncertainty prevents us from a discussion of the saturation ratio in detail, and emphasizes that accurate temperature measurements are important to determining accurate saturation ratios. Nevertheless, from Figure 14, high super‐saturations beyond this large uncertainty could be inferred where the temperature is very cold (<150 K). These very cold regions correspond to the high latitudes at equinox seasons (Figures 14a, 14c, and 14o) and high‐altitude regions (>60 km, reddish points in Figure 14). At these low temperature regions, water saturation pressures are down to ∼10^−5^–10^−6^ [Pa]. Such low partial pressures can be exceeded with the presence of only several ppm of water vapor, which are observed by NOMAD.

## 7 Conclusions

We have analyzed 3.5 years of daily solar occultation measurements by TGO/NOMAD. It reveals the new details of global vertical distribution of water vapor on Mars. The main results are as follows.

- Shortly after equinox periods, water vapor is confined to the middle‐low latitudes (±∼50°). The water vapor abundances and their vertical extent are larger in the case of the northern autumn equinox than the vernal equinox. The maximum volume mixing ratio reaches ∼210 ppm and vertical extent up to ∼40–50 km shortly after the northern autumn equinox, and it is below ∼80 ppm below 30–40 km shortly after the northern vernal equinox. The water vapor vertical distribution is asymmetric around the equator, in that it is more pronounced in the spring hemisphere. The polar regions are very dry and cold, where a large super‐saturation is inferred.
- In aphelion periods, water vapor sublimated from the northern polar cap is confined to very low altitudes (below 10–40 km) with an order of magnitude reduction in mixing ratio by ∼15 km at the high‐latitudes (>50°N) and by ∼30 km at the middle‐latitudes (30–50°N). The vertical extent at the middle latitudes is more pronounced in the evening terminator than morning. It suggests that the diurnal cycle of the water ice clouds associated with thermal tides controls the vertical extent of water vapor. Water vapor is also present over equatorial regions in the southern hemisphere mostly below 10–20 km, which indicates north‐south water transport still occurs.
- In perihelion periods, water vapor sublimated from the southern polar cap directly reaches high altitude (at least 80 km). It is attributed to the intense perihelion meridional circulation, which transport water without condensing due to higher atmospheric temperature. Large amounts of water vapor (>100 ppm) are widely present in the middle atmosphere except over the northern high latitudes. At the end of perihelion period, abundance of water vapor in the middle atmosphere decreases gradually. However, it rapidly increases again around *L*_S_ = 320° due to the annual strong regional dust storms.
- We confirm the strong contrast between aphelion and perihelion water climate. We show that the main events/locations to supply water vapor to the upper atmosphere above 70 km are (a) southern high latitudes at perihelion periods due to intense meridional circulation, and (b) dust storms (sporadic global dust storms and annual regional dust storms around *L*_S_ = 320°).

## Data Availability

The results retrieved from the NOMAD measurements used in this article are available at https://doi.org/10.18758/71021072 (Aoki & Vandaele, 2022). The NOMAD data used in this study are available from ESA's Planetary Science Archive at https://archives.esac.esa.int/psa/%23%21Table%20View/NOMAD%3Dinstrument.
